# Annotations

**Published:** 1906-10-06

**Authors:** 


### ANNOTATIONS.

### "The Wellcome Research Laboratories."

We have recently received the second Report of the Wellcome Research Laboratories at the Gordon Memorial College, Khartoum. Dr. Andrew Balfour, the director, is responsible for its production, and he certainly must be congratulated, not only for the excellent work contained within its pages, but also for the excellent way in which he has got it up. The report is handsomely bound, printed on fine paper, and profusely illustrated with lovely coloured diagrams, many from the pen of Mr. Perzi, as well as with the ordinary black-and-white sketches and photographs. Such illustrations, of course, enhance the value of the report enormously. Again, Dr. Balfour has been fortunate in enlisting the co-operation of Mr. Austen, of the British Museum, and Mr. Theobald, of Wye, in contributing chapters on their own special subjects on material collected by him in the Soudan. For example, the former has a chapter entitled "On some Blood-sucking Diptera from the Anglo-Egyptian Sudan, collected during the year 1905, with Descriptions of New Species," while the latter writes a "Second Report on the Mosquitoes or Culicidæ of the Sudan" and chapters on "Human, Animal, and Vegetable Pests," all subjects of great interest. In addition to those items Dr. Balfour publishes his own work, to which we shall refer later; and then there are also reports by Dr. Sheffield Leave, the travelling pathologist and naturalist; and Dr. Beam, in charge of the chemical department. Such an accumulation of material by well-known authorities commands attention and perusal. The quality of the work is of a very high class, and some of it is quite new to science. The hœmogregarine of the Jerboa, H. Balfouri (Laveran), is of exceptional interest, as parasites of that nature have never been found in the red blood-corpuscles of mammals before. In addition to making this important discovery, the author has followed out the further stages of the parasite in the liver of affected animals, and believes he has found its extra-corporeal cycle in fleas. Dr. Neave's contribution contains some very interesting notes on parasites of birds and other animals, quite a number of new filariæ having been discovered in the former. A careful study of this very excellent Report will well repay the reader.

### The Grantham Disaster.

The evidence placed before the coroner's jury investigating the Grantham railway disaster has been insufficient to enable them to return any but an open verdict, and while it is unsatisfactory that they have been unable to assign the lamentable disaster to a well-established fact, the verdict is so far satisfactory in showing that, with the single doubtful exception of the open points, there was no evidence of weakness in engine, carriages, or permanent way. In other words, the mechanical part of the machinery seems to have been irreproachable. On the other hand, a scrutiny of the evidence leaves little doubt that the fault lay with the human element. To this conclusion that large and vociferous jury, the British public, had already come. Many bizzare theories have been promulgated to account for the driver's failure to shut off steam and apply his brakes—madness, forgetfulness, illness, death, and even a fatal quarrel. Of all the suppositions brought forward to account for the mistake lapse of memory alone strikes us as worthy of a moment's credence. Narrow escapes from disaster arising from a moment's forgetfulness are probably commoner than is thought. The human element in machinery is frequently unreliable. Employers of labour aver that not only can a piece of mechanism work more rapidly and more cheaply than man, but that the machine is the more reliable of the two. It never forgets. Trains cannot be driven solely by machinery. It is the duty of the public to consider how it is possible to strengthen this weak link. Express trains should have more than two men on the foot-plates. The engine pulling the Grantham train was one of the largest engines in the country. To put a giant of this kind under the charge of only two men, when on a long-distance journey, seems to the ordinary individual the way to court disaster.

### The Practice of Ophthalmology.

In an able paper contributed to the *Ophthalmic Review* Dr. Freeland Fergus advocates the establishment of a special training, a special diploma, and a special register in connection with ophthalmic medicine and surgery. He would not excuse the would-be ophthalmic surgeon from the ordinary medical curriculum, nor would he interfere with the liberty of any qualified medical practitioner to treat cases of eye disease. But he does desire a special ophthalmic training and diploma as a condition precedent to the entry of any person's name on the ophthalmic register. Apparently the plan contemplates the deliberate adoption of special practice from a very early date in the student's career, as the examination preliminary to the student's training ought, according to Dr. Fergus, to include the elements of the calculus and plane trigonometry. With all this we must confess we have little sympathy. The practice of ophthalmology cannot with safety or advantage be separated from general medicine and surgery, and anything which tends to mark it off as a special preserve is, in our judgment, contrary to the best interests and traditions of the profession. Indeed, what is wanted is an influence exactly in the opposite direction, namely, a proclamation of the fact that the pathological conditions which occur in the eye can be readily recognised by any practitioner who is willing to spend a little time and trouble, and that the recognition of these conditions when they exist has the closest possible association with general diagnosis and treatment. Doubtless many of the surgical operations on the eyeball require a delicacy of manipulation which can only be secured by those who are frequently undertaking them, and here certainly is room for special practice. But in its general relations ophthalmology belongs to the common practice of the profession, and the more fully this is insisted on the better for the suffering public.

